# Liquid Phase Hydrogenation of Pharmaceutical Interest Nitroarenes over Gold-Supported Alumina Nanowires Catalysts

**DOI:** 10.3390/ma13040925

**Published:** 2020-02-19

**Authors:** Krishnamoorthy Shanmugaraj, Tatiana M. Bustamante, Cristian H. Campos, Cecilia C. Torres

**Affiliations:** 1 Departamento de Físico-Química, Facultad de Ciencias Químicas, Universidad de Concepción, Edmundo Larenas 129, Concepción 4070371, Chile; shanmugaraj.chemist@gmail.com (K.S.); tatibustamante@udec.cl (T.M.B.); ccampos@udec.cl (C.H.C.); 2 Departamento de Química, Facultad de Ciencias Exactas, Universidad Andres Bello, Sede Concepción, Autopista Concepción-Talcahuano 7100, Talcahuano 4300866, Chile

**Keywords:** nanowires, gold, nitroarenes, Aluminium oxide

## Abstract

In this work, Au nanoparticles, supported in Al_2_O_3_ nanowires (ANW) modified with (3-aminopropyl)trimethoxysilane were synthetized, for their use as catalysts in the hydrogenation reaction of 4-(2-fluoro-4-nitrophenyl)-morpholine and 4-(4-nitrophenyl)morpholin-3-one. ANW was obtained by hydrothermal techniques and the metal was incorporated by the reduction of the precursor with NaBH_4_ posterior to superficial modification. The catalysts were prepared at different metal loadings and were characterized by different techniques. The characterization revealed structured materials in the form of nanowires and a successful superficial modification. All catalysts show that Au is in a reduced state and the shape of the nanoparticles is spherical, with high metal dispersion and size distributions from 3.7 to 4.6 nm. The different systems supported in modified-ANW were active and selective in the hydrogenation reaction of both substrates, finding for all catalytic systems a selectivity of almost 100% to the aromatic amine. Catalytic data showed pseudo first-order kinetics with respect to the substrate for all experimental conditions used in this work. The solvent plays an important role in the activity and selectivity of the catalyst, where the highest efficiency and operational stability was achieved when ethanol was used as the solvent.

## 1. Introduction

The catalytic hydrogenation of nitroarenes (-NO_2_) is one of the most important organic reactions for the preparation of amines (-NH_2_), since they are used as intermediates or precursors in the synthesis of drugs, agrochemicals, dyes and other organic compounds [1,2,3,4,5]. The traditional methods for preparing these compounds involve the use of dangerous reducing agents and/or contaminants, such as N_2_H_4_, Na_2_S, silyl hydrides, in situ H_2_ generation, among others [2,6]. For example, the used of direct metal produces H_2_ by reacting with water or by electron-transfer reaction with water acting as proton source. Hydrazine hydrate methodologies release H_2_ by decomposition facilitating the reduction process. However, these strategies present notable disadvantages such as high reaction temperatures, poor chemoselectivity over other functional groups and lower yield to the desire product due to a mixture of products [7,8]. In this context, heterogeneous catalysis is an alternative to these procedures, because these reactions can be highly selective, efficient, environmentally friendly and cheaper, since the catalyst can often be recovered and recycled [2]. In addition, it is noteworthy that mild experimental conditions can be modulated in the hydrogenation reaction at lower or atmospheric pressure depending on the catalyst and the nitroarene [9,10].

Gold (Au) as an active phase has shown to be the most selective metal in the hydrogenation of multifunctional nitroarenes [11,12,13,14,15,16,17,18]. Nevertheless, one of the main challenges in the preparation of Au-based catalysts has been the control of particle size to generate active, selective and operationally stable systems in the hydrogenation of nitroarenes [19]. Some strategies consider the preparation of nanometric catalysts, obtained from core-shell (0D) structures that include in their design the insertion of the active phase in the core [20,21,22] or the Shell [23]. Other reported strategies include the preparation of supported catalysts by the deposition of Au in support via 1D arrangements (nanotubes, nanowires) [18,24,25,26], 2D (graphene sheets, clays) [27,28,29], or in 3D structures such as mesostructured silicas [16,30,31], carbonaceous supports [32,33,34] and others. 

Alumina is widely used as a heterogeneous catalyst support due to its attractive proprieties mainly due to their high mechanical and thermal stability [35,36]. The use of γ-Al_2_O_3_ as a nanosupport with high mesoporosity is considered one of the most efficient strategies to achieve efficient mass transport and, therefore, high catalytic performance in heterogeneous catalysis [37,38,39,40]. One of the simplest and most reproducible methodologies to obtain γ-Al_2_O_3_ with hierarchical porosities is reported by Lu et al., where it is possible to obtain nanowires (NW) with high mesoporosity and specific surface areas (S_BET_) in the order of 200 m^2^g^−1^ [41]. 

**Scheme 1:**
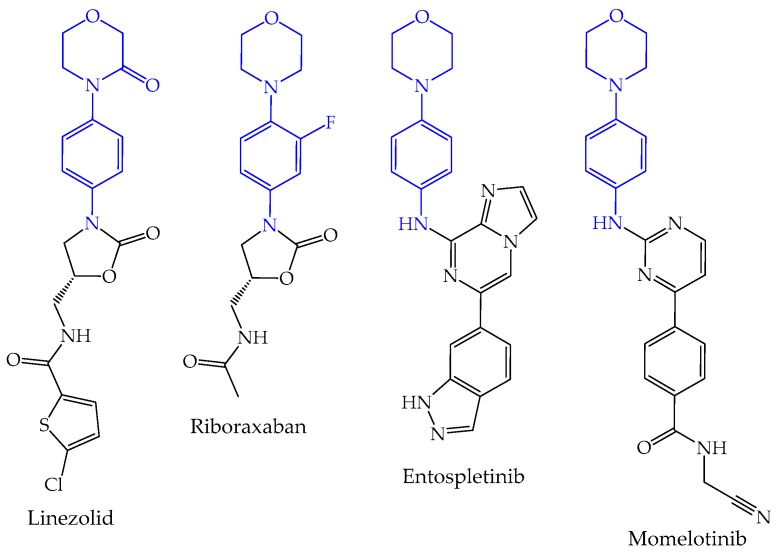
The 4-morpholinoaniline derivate drugs: the antimicrobial Linezolid (Zyvox), the anticoagulant blood thinner Rivaroxaban (Xarelto) and the investigational antineoplastic drugs Entospletinib and Momelotinib.

As mentioned, nitrocompounds are building blocks for many products of industrial interest and gold supported Al_2_O_3_ catalyst has been applied for different nitroarenes hydrogenation. Wang et al. reach high selectivity for *p*-chloronitrobenzene and Yakushkin et al. obtained chemoselective hydrogenation of 3-nitrostyrene and 4-nitroacetophenone [42,43,44]. However, more complexes nitroarenes such as 4-(4-nitrophenyl)-morpholine derivates (see Scheme 1) are mostly obtained through traditional synthesis pathways and did not report kinetic data [45]. Only a few report exploring molecules with pharmaceutical applications has been published. Gardiner et al. used catalytic static mixers coated with Pd to obtain 3-fluoro-4-morpholinoaniline (Yield_prod_ < 30%) [46]. Bustamante et al. employ Pd-Co catalysts to study a series of halonitroarenes under soft hydrogenation conditions (ethanol as solvent, 80 °C and 20 bar of H_2_ pressure) with selectivity to the desired aniline greater than 95% [47].

The focus of this work is on the synthesis, characterization and catalytic evaluation of nanocatalysts prepared from the deposition of Au nanoparticles in supports of γ-Al_2_O_3_ type NW (ANW), to be evaluated in the chemoselective hydrogenation of nitroarenes of pharmaceutical interest. The catalysts were prepared in different nominal metal loadings: 0.25, 0.50 and 1.00 Au wt%. All the catalysts were evaluated in the catalytic hydrogenation of 4-(2-fluoro-4-nitrophenyl)-morpholine (NFMF) and 4-(4-nitrophenyl)-3-morpholinone (NFMO) used as intermediates in the synthesis of linezolid (antibiotic action) and rivaroxaban (anticoagulant action), respectively [48,49]. In both molecules, different operational conditions were evaluated to produce the respective aren-anilines. For the best catalyst, operational stability was evaluated under optimized hydrogenation conditions. This report presents for the first time the hydrogenation reaction of pharmaceuticals interest molecules and reutilized Au/ANW as a new alternative to produce reactions intermediates of drugs.

## 2. Materials and Methods

### 2.1. Materials

Al(NO_3_)_3_·9H_2_O, (1R)-(−)-camphorsulfonic acid (CSA, 98%), (3-Aminopropyl)trimethoxysilane (APTMS, ≥98%), morpholine, N,N-diisopropylethylamine, acetonitrile, methanol, toluene, absolute ethanol, ethyl acetate, dichloromethane, chloroform, tetrahydrofurane (THF), hexane, HNO_3_ (67%), and HCl (37%), Na_2_SO_4_ anhydrous, potassium permanganate, sodium bisulfite, aqueous ammonia solution (NH_4_OH 37%) and HAuCl_4_·3H_2_O were provided by Merck (Darmstadt, Germany). Benzyltrimethylammoniun chloride and 4-phenylmorpholine were provided by Sigma-Aldrich (Darmstadt, Germany). Toluene was dried over metallic sodium before use. All other reagents were used without further purification. H_2_ (99.99%), He (99.99%), synthetic air, and N_2_ were provided by Linde Chile (Concepción, Chile).

### 2.2. Synthesis of the Substrates

The drug precursors 4-(2-fluoro-4-nitrophenyl)morpholine and 4-(4-nitrophenyl)morpholin-3-one could not be sourced from commercial suppliers, so they were synthesized and characterized as detailed.

#### 2.2.1. Synthesis of 4-(2-fluoro-4-nitrophenyl)morpholine

3,4-difluoronitrobenzene (3.0 mL, 4.31 g, 27.1 mmol) was added to a mixture of morpholine (4.72 mL, 4.72 g, 54.2 mmol) and N,N-diisopropylethylamine (9.44 g, 54.2 mmol) in acetonitrile (27 mL), and the resulting mixture was stirred at room temperature for 48 h. The final solution was dried and dissolved in ethyl acetate. The product was washed with water (3×) and brine. The organic layer was separated and dried over Na_2_SO_4_, filtered, and concentrated under vacuum to give a yellow solid (6.32 g, ca. 98%). ^1^H NMR (400 MHz, CDCl_3_) δ: 7.93 (1H, d, J = 7.5 Hz), 7.84 (1H, d, J = 10.5 Hz), 6.89 (1H, t, J = 8.0 Hz), 3.84 (4H, t, J = 5.0 Hz), 3.25 (4H, t, J = 9.5 Hz) ppm. ^13^C NMR (75 MHz, CDCl_3_) δ: 50.0, 66.7, 112.7, 117.0, 121.1, 140.8, 145.6, 152.2–154.2 ppm.

#### 2.2.2. Synthesis of 4-(4-nitrophenyl)morpholin-3-one

**Scheme 2:**
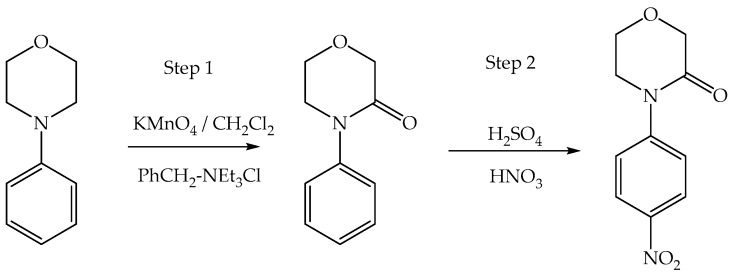
Synthetic pathway for the preparation of 4-(4-nitrophenyl)morpholin-3-one.

The synthesis of 4-(4-nitrophenyl)morpholin-3-one was carried out by two steps of synthesis as shown in Scheme 2. In step one, the intermediary was prepared following synthesis, as reported by Markgraf et al. [50]. Finely ground potassium permanganate (0.474 g, 3.00 mmol) was added to a stirred solution of the 4-phenylmorpholine (0.326 g, 2.00 mmol) and benzyltrimethylammoniun chloride (0.683 g, 3.00 mmol) in dichloromethane (10 mL), and the purple solution was stirred at reflux for 3 h.

The mixture was cooled in ice and a solution of sodium bisulfite (2 g) in water (10 mL) was added and separated; the aqueous phase was extracted with chloroform (3 × 10 mL). The combined organic extract was washed with water (20 mL) and dried with Na_2_SO_4_. The solvent removal gave a residual yellow solid (0.205 g, 56% yield) which was recrystallized from hexane to give 4-phenylmorpholin-3-one (0.135 g, 35% yield). ^1^H NMR (400 MHz, CDCl_3_) δ 7.41 (2H, m). 7.31 (3H, m), 4.34 (2H, s), 4.02 (2H, app t, J = 5.0 Hz), δ 3.76 (2H, app t, J = 5.0 Hz) ppm. ^13^C NMR (75 MHz, CDCl_3_): δ 49.7, 64.2, 68.6, 125.6, 127.2, 129.4, 141.4, 166.7 ppm.

The second step was carried out following the experimental methodology reported by Xing et al. [49]. To a solution of 4-phenylmorpholin-3-one (0.995 g, 5.62 mol) at 98%, H_2_SO_4_ (2.33 mL) cooled to −10 °C 65% HNO_3_ was carefully added dropwise (0.4 mL) over 45 min. The reddish solution obtained was stirred at this temperature for 1 h and H_2_O (5 mL) was added. The mixture was neutralized with 35% NH_4_OH until pH 7. The resulting precipitate was filtered, washed with cold water and dried, resulting in the desired product (1.01 g, 83% yield) as a brown solid. ^1^H NMR (400 MHz, CDCl_3_) δ: 8.27 (2H, d, J = 9.4 Hz,), 7.62 (2H, d, J = 9.4 Hz), 4.38 (2H, s), 4.08 (2H, t, J = 5.0 Hz), 3.85 (2H, t, J = 5.0 Hz) ppm. ^13^C NMR (75 MHz, CDCl_3_): δ 49.6, 64.2, 68.6, 124.2, 125.6, 145.4, 146.4, 167.4 ppm.

### 2.3. Al_2_O_3_ Nanowires (ANW) and Catalysts Synthesis

The synthesis of the ANW was carried out by a method previously reported by our research group with some modifications [51]. Twenty-two grams of Al(NO_3_)_3_·9H_2_O were dissolved together with 2.2 g of CSA as a surfactant, in 160 mL of distilled H_2_O, and kept under magnetic stirring. The mixture was brought to pH 5.4 using NH_4_OH, forming a white gel which was kept under stirring for 10 min. Subsequently, the gel was transferred into a stainless-steel autoclave coated with teflon and held for 72 h at 160 °C. It was then allowed to cool to room temperature and the material was centrifuged, then washed repeatedly with absolute ethanol and dried in an oven at 100 °C. Finally, the material was ground, sieved at 350 mesh and calcined at 550 °C for 4 h, in a synthetic-air flow of 20 mL min^−1^ to obtain a white solid labelled ANW.

To promote the homogeneous dispersion of the Au nanoparticles onto the ANW surface, the support was modified with the coupling agent APTMS by methodologies reported by Dinamarca et al. [52]. In a two-necked, round-bottom flask, 100 mL of dry toluene, 5.0 g of ANW and 5 mL of APTMS were dispersed and stirred for 1 h. The system was refluxed for a period of 24 h in inert atmosphere. The modified solid was centrifuged and washed with toluene (1 × 50 mL) and ethanol (2 × 50 mL) and dried in a vacuum oven for 12 h at 60 °C. The obtained solid was labelled as ANW-NH_2_.

Three catalysts were prepared at nominal Au loadings of 0.25, 0.50 and 1.0 wt%. The synthesis was performed by dispersing 1.0 g of the ANW-NH_2_ support in 10 mL of methanol. The solid was sonicated for 10 min and, subsequently, the necessary amount of solution, 5.16 mM HAuCl_4_ in methanol, was added to the dispersion, which was maintained under stirring for a period of 4 h. Then, 2 mL of an aqueous solution of 0.1 M NaBH_4_ was added. Subsequently, the system was kept under stirring for a period of 30 min. Finally, the dispersion was centrifuged and washed with water until it reached a neutral pH. The catalysts were labelled as (x)Au/ANW, where x corresponds to the nominal metal loading in the catalyst formulation. 

### 2.4. Characterization

Chemical composition was probed by atomic absorption spectrometry (AAS) employing a Perkin Elmer 3100 instrument (Perkin Elmer, Manasquan, NJ, USA). The samples were evaluated in triplicate by microwave-assisted digestion of 0.05 g of the catalyst in 10 mL of concentrated HNO_3_/HCl dissolution (1:3, v/v). Solid-state ^13^C and ^29^Si cross-polarization (CP)-magic angle spinning (MAS) NMR spectra were recorded at 100.6 MHz and 79.49 MHz, respectively, using a Bruker AV 400 WB spectrometer (Bruker, Rheinstetten Germany). Specific surface areas were calculated using the Brunauer–Emmett–Teller (BET) method from N_2_ adsorption–desorption isotherms, recorded at −196 °C using a Micromeritics TrisStarII 3020 instrument (Micromeritics, Norcross, GA, USA). X-ray powder diffraction (XRD) patterns were recorded using Ni-filtered Cu *K*_α__1_ radiation (*λ* = 1.5418 Å) on a Rigaku diffractometer (Rigaku, Tokyo, Japan), in a 2*θ* range of 20–90°. Transmission electron microscopy (TEM) micrographs were obtained using a JEOL model JEM-1200 EX II microscope (JEOL, Tokyo, Japan). X-ray photoelectron spectra (XPS) were recorded using an Escalab 200 R spectrometer (Thermo Scientific, Waltham, MA, USA) equipped with a hemispherical analyser and using non-monochromatic Mg K_α_ X-ray radiation (hυ = 1253.6 eV). The spectra were fitted to a combination of Gaussian–Lorentzian lines of variable proportion. The C 1s core-level of adventitious carbon at a binding energy (BE) of 284.8 eV was used as an internal standard.

### 2.5. Catalytic Activity

The catalytic activity of the Au-supported catalyst for the nitroarene hydrogenation was evaluated using 0.020 g catalyst samples and the amounts of substrate necessary to obtain a mol_substrate_:mol_Au_ ratio of 100, based on the metal contents determined by AAS. All activity measurements were performed in triplicate using a semi-batch Parr^®^-type reactor with magnetic agitation filled with absolute ethanol as solvent (50 mL) at an H_2_ pressure of 20 bar and 75 °C. Products were identified by gas chromatography using a GC Hewlett Packard HP 4890 D gas chromatograph (Hewlett Packard HP-4890, Hewlett Packard, Palo Alto, CA, USA) equipped with an HP-5 capillary column and a flame-ionization detector. Conversion (*X*_substrate_) and selectivity (*S*_product_) were calculated based on calibration curves and the following equations:

(1) $$X_{subtrate}\left(\%\right)=\frac{\left({\left[substrate\right]}_{i}-{\left[substrate\right]}_{t}\right)}{{\left[substrate\right]}_{i}}\times 100$$

(2) $$S_{product}\left(\%\right)=\frac{\left({\left[product\right]}_{t}\right)}{\left({\left[substrate\right]}_{i}-{\left[substrate\right]}_{t}\right)}\times 100$$

where i corresponds to the concentration at the initial time (*t* = 0) and t is the concentration at any time different to the initial (*t* > 0).

To confirm the presence of byproducts, the reactions were monitored using a gas chromatograph coupled to a mass detector Perkin Elmer GCMS-SQ8T (Perkin Elmer, Manasquan, NJ, USA). For the solvent effect studies, the catalytic measurements were carried out employing the same reaction conditions, but the reactor was filled with acetonitrile, methanol, toluene, ethyl acetate or THF as solvents. Catalyst operational stability was probed by recycling tests. The catalyst was separated by hot filtration from its bed, washed three times with the reaction solvent and sequentially treated with 50 mL of fresh solvent and the amount of substrate required to repeat the reaction. This operation was repeated five times in succession. 

## 3. Results and Discussion

### 3.1. Support and Catalysts Characterization

**Figure 1:**
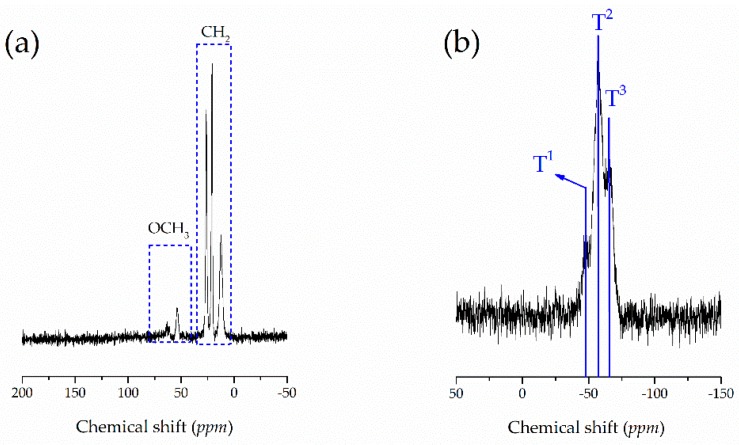
Solid state cross-polarization (CP)-magic angle spinning (MAS) NMR of the Al_2_O_3_ nanowires (ANW) modified with (3-Aminopropyl)trimethoxysilane (APTMS). (**a**) ^13^C and (**b**) ^29^Si.

The incorporation of the APTMS functionality within the ANW support was confirmed by the solid-state NMR spectra. As shown in Figure 1a, the ^13^C magic angle spinning (MAS) NMR spectra of the supports presented one group of typical signals at 13.3, 20.3 and 26.6 ppm, corresponding to CH_2_ groups from propyl-moiety, and signals detected at 50–60 ppm, which correspond to the C from Si-OCH_3_ functional groups. In addition, the ^29^Si cross-polarization (CP) MAS NMR spectra demonstrated the anchorage of APTMS on the ANW surface. As shown in Figure 1b, the NMR spectra of the ANW-NH_2_ displayed typical signals distributed broadly from −45 to −70 ppm, attributed to the presence of organic molecules with silyl ether groups. The typical isomer shift values in the literature for T^1^, T^2^ and T^3^ signals at −48.5, −58.5 and −67.5 ppm, respectively [53]. In our case, the main signals appears for the T^2^–T^3^ type of anchorage that is attributed to the grafting reaction between the surface Al–OH groups and silane moieties to produce (Al-O)_x_-Si-(OCH_3_)_3−x_(CH_2_CH_2_CH_2_-NH_2_) with x = 1 and/or 2. This result is in line with the C signals detected at 50–60 ppm in NRM ^13^C spectrum, which correspond to the unreacted methoxide–silyl ether groups. 

AAS characterization was carried out to quantify the Au content in the catalysts, as shown in Table 1. The similarity of experimental and nominal metal contents indicated the absence of appreciable metal loss during synthesis of the catalysts.

**Figure 2:**
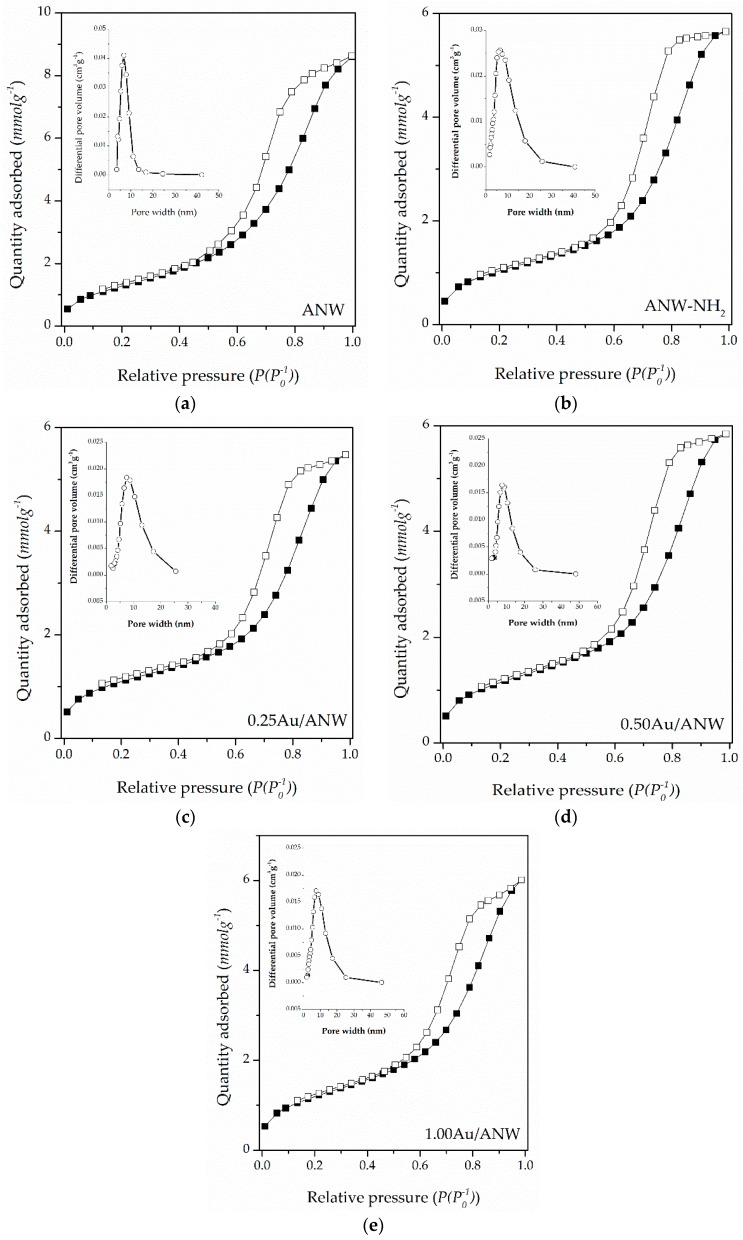
N_2_ adsorption–desorption isotherms and pore-size distribution curves of the prepared materials. (**a**) ANW; (**b**) ANW-NH_2_; (**c**) 0.25Au/ANW; (**d**) 0.50Au/ANW; (**e**) 1.00Au/ANW. Close square corresponds to adsorption branch and open square corresponds to the desorption branch.

Figure 2 displays the adsorption–desorption isotherms of N_2_ at −196 °C for the as-synthetized materials. The analysis of isotherms and S_BET_ (Table 1) shows that all the solids were mesoporous and had a type IV, according with to IUPAC classification, and possess hysteresis cycle type H1, corresponding to cylindrical pores. Moreover, all of them displayed monomodal mesopore-size distributions. This information allows us to infer that, as the Au loading increased on the supports, the values of surface area, pore volume, and pore size dropped, due to the anchoring of APTMS and the metallic nanoparticles on the surface, respectively.

The characterization by XRD of the supports and catalysts prepared is shown in Supplementary Material (Figure S1). In all cases, the typical diffractions for γ-Al_2_O_3_ (JCPDS 86–1410) were detected. The solid maintains the diffraction pattern for the support after the APTMS immobilization treatment. Differences attributed to metallic Au are not detected, as the diffractions for Au (111) at 2θ = 38.1° and (200) at 2θ = 45.0° are masked by the diffractions of the ANW support.

**Figure 3:**
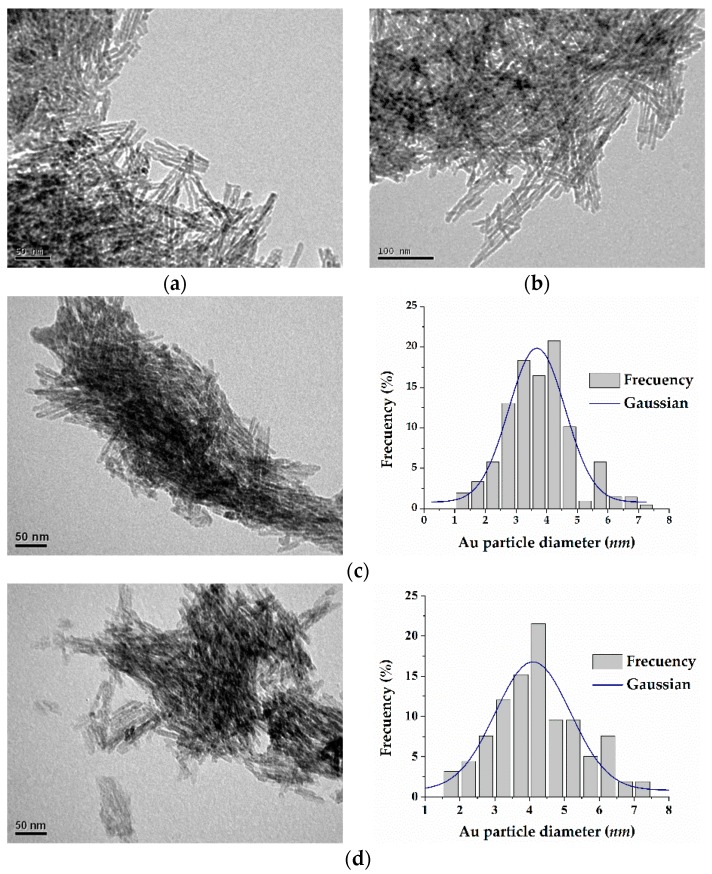

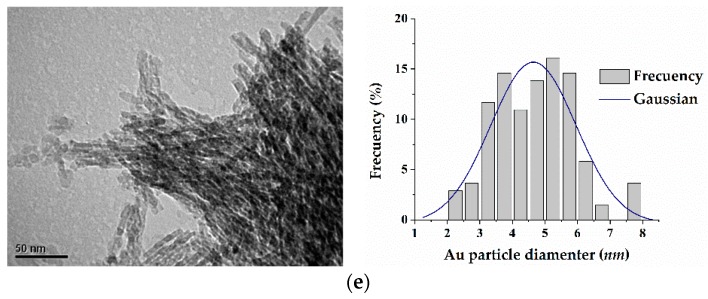
TEM micrographs and Au NPs size distribution of the as-synthesized supports and catalysts. (**a**) ANW; (**b**) ANW-NH_2_; (**c**) 0.25Au/ANW; (**d**) 0.50Au/ANW and (**e**) 1.00Au/ANW.

The micrographs of TEM for ANW, ANW-NH_2_ and the catalysts (x)Au/ANW are shown in Figure 3. The morphology of pristine and modified nanowires is confirmed and no change in morphology is observed after metal deposition. ANW support was modified on its surface with APTMS to promote the uniform deposition of metal nanoparticles once they are deposited in the material. According with our previous reported results, the use of APTMS enhanced the uniform distribution of the metal precursor on the nanomaterials surface [52]. This behaviour is attributed to a ligand exchange between chlorine (Cl^−^) groups of AuCl_4_^−^ metal precursor and amine (NH_2_) groups from the immobilized APTMS. The HAuCl_4_ can exchange the chloride ligand with NH_2,_ providing the formation of mixed amine-chloro complexes [AuCl_4−x_(NH_2_)_x_]^x−1^, as has been reported by Lei et al. [54]. According with TEM characterization, all the (x)Au/ANW catalysts showed Au nanoparticles with narrow particle size distributions. The average size of the nanoparticles increases as the metallic content of Au in the catalysts increases, as shown in Table 1. We suggest that the increase in the metal loading promotes the Au NPs’ agglomeration following the Ostwald ripening process during the reduction process with NaBH_4_. 

**Figure 4:**
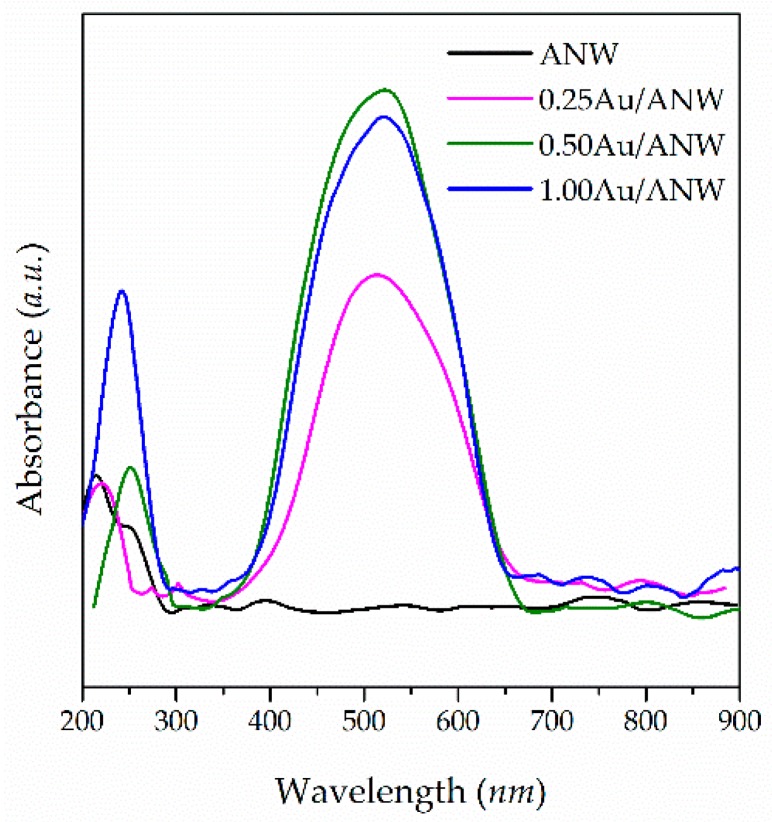
UV−vis−NIR diffuse reflectance spectroscopy (DRS) of ANW support and (x)Au/ANW catalysts.

Figure 4 show the characterization of the catalysts by UV−vis−NIR diffuse reflectance spectroscopy (DRS). Pristine ANW did not show any contribution absorbance band in the visible wavelength range. All the catalytic systems showed a broad surface plasmon resonance (SPR) adsorption peak centred at 524 nm [55]. In addition, all the catalytic system showed a second absorption band at 250–260 nm which is characteristic of cationic gold species [56,57]. An increase in the contribution of cationic Au species with the increase of Au loading was observed. This trend could be attributed to the incomplete reduction of metal-precursor during the treatment with NaBH_4_. According with N_2_ isotherms and TEM characterization, the increase of Au metal particles size with the increase of metal loading produced a pores blockage; this occlusion could be inhibit the reduction of the sorbed [AuCl_4−x_(NH_2_)_x_]^x−1^ species into the pores structures.

**Figure 5:**
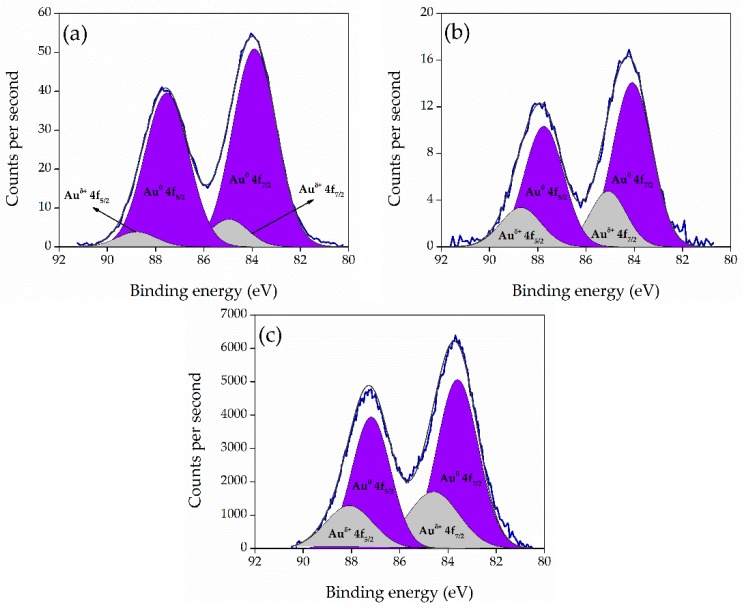
X-ray photoelectron spectra (XPS) spectra of Au 4f for the as-synthetized (x)Au/ANW catalysts. (**a**) x = 0.25; (**b**) x = 0.50 and (**c**) x = 1.00.

To confirm surface electronic states and the chemical composition of the prepared system, all the catalysts were examined by using X-ray photoelectron characterization (XPS), as shown in Figure 5. The binding energies (BE) of the Au 4f_7/2_ states for Au/ANW catalysts are located at 83.5–83.9 eV (see Table 2), which agree with the values reported in the literature for metallic Au [12,13,18]. In addition, some cationic species with Au 4f_7/2_ signals located at 84.5–85.1 eV [58], respectively, are also detected.

This behaviour is agreement with the DRS UV-Vis characterization results. The presence of Au^δ+^ species after reduction treatment was attributed to an incomplete reduction of the metal-precursor during the catalyst synthesis. Moreover, it should be noted that, as the Au content decreases, Au 4f_7/2_ BE shifted from 83.9 eV for 1.00Au/ANW catalyst, to 83.4 eV, in the case of 0.25 Au/ANW. This kind of shift in BE is very common for very small gold particles supported in Al_2_O_3_ supports, due to reduced core-hole screening in small metal particles [44]. This indicates that there are significant differences in the electronic properties of very small particles and bulk materials and that the dimensionally dependent changes in the electronic structure produce unusual characteristics [40,59,60]. 

### 3.2. Catalytic Activity 

#### 3.2.1. Effect of Au Loading 

**Figure 6:**
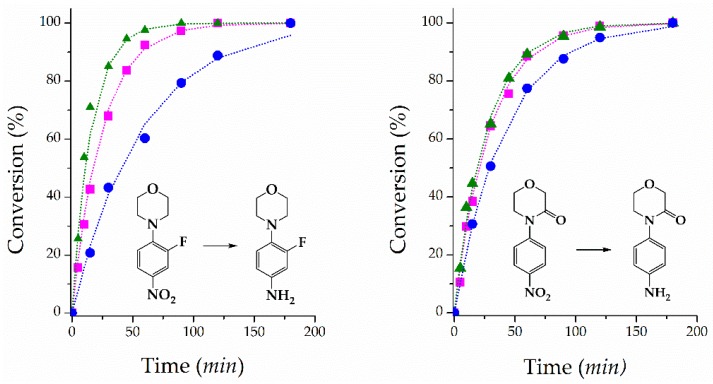
Conversion curves as a function of time for hydrogenation of the substrates of pharmaceutical interest on the catalysts (x)Au/ANW. ■—0.25Au/ANW; ▲—0.50Au/ANW and ●—1.00Au/ANW. Straight lines correspond to the kinetic pseudo-first order adjustment.

**Scheme 3:**
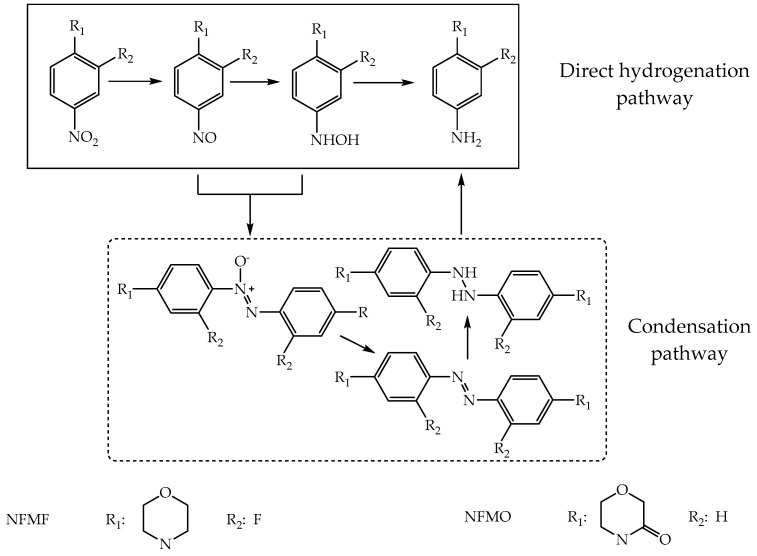
Reaction pathways for nitroarenes’ hydrogenation.

The catalytic activities of the (x)Au/ANW catalysts are shown in Figure 6. The kinetics measurements revealed that the supported Au catalysts exhibited a high efficiency in both the NFMF and the NFMO hydrogenation reactions, showing a pseudo-first order kinetics, with respect to the substrate consume. During the reaction course, the formation of the corresponding anilines (>99%) was highly favoured over the accumulation of reaction intermediates (Scheme 3), showing that the (x)Au/ANW systems are very selective catalysts for nitroarenes hydrogenation.

These findings are in agreement with the experimental conclusions reported by Torres et al. on a TiO_2_ nanotubes-supported gold catalyst [18]. A relevant outcome regarding these results is that high catalytic activity and high selectivity towards hydrogenation to anilines were achieved by the (x)Au/ANW systems, despite their low metal content. In this context, our catalyst shows better performance than previous reports from Gardiner et al. that used Pd immobilized to hydrogenated NFMF reaching to 30% of yield to the amine-arene desire [46]. With respect to selectivity, one intermediate reaction was detected, namely N-(substituted-phenyl)-hydroxylamine derivate (<1%), with no evidence of condensation byproducts. Both intermediates were detected at very low concentrations, thus supporting the predominance of a direct hydrogenation route over a condensation route, as shown in Scheme 3. To confirm our results, two control experiments were performed: catalytic reaction in the absence of hydrogen to rule out the reduction of both nitroarenes by hydrogen transfer using ethanol as H donor and the reaction in the absence of the catalyst. In all experiments, no conversions greater than 5% were detected at 24 h of operation. 

Regarding the effect of the metallic charge, a direct dependence of the reaction rate is observed as a function of the metal loading. In the case of 1.00Au/ANW catalyst showed the largest particle size and the lowest conversion compared to the systems with lower metal content. The decrease of catalytic performance could be attributed to the Au metal mean particle size. The presence of Au nanoparticles with d_TEM_ = 4.6 ± 2.6 nm on the 1.00Au/ANW catalyst surface often results in reduced activity of the catalyst due to diffusion limitations and/or blocking of active sites. In the case of 0.25Au/ANW and 0.50Au/ANW, both catalysts have a similar particle size, the 0.25Au/ANW catalyst has less Au availability on the surface compared to 0.50Au/ANV, as shown in the characterization by XPS, as shown in Table 2. 

#### 3.2.2. Effect of the Solvent

**Figure 7:**
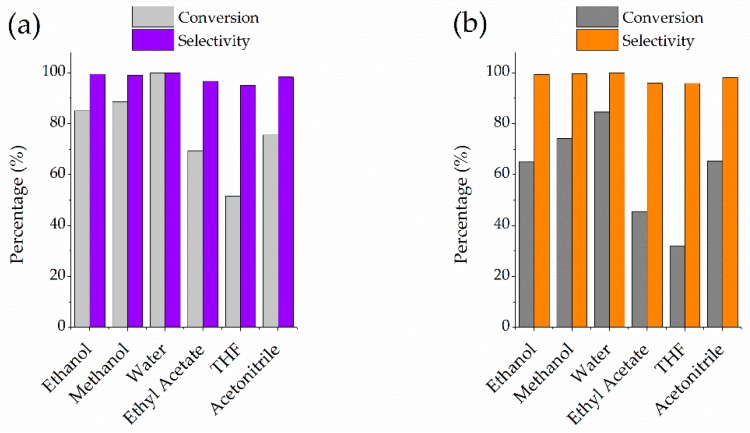
Effect of the solvent on the activity and selectivity in the hydrogenation of the selected nitroarenes at 30 min of reaction. (**a**) 4-(2-fluoro-4-nitrophenyl)-morpholine (NFMF) and (**b**) 4-(4-nitrophenyl)-3-morpholinone (NFMO).

The best activity results were shown by the catalyst 0.50Au/ANW, in which different solvents were evaluated. The selected solvents were ethanol, methanol, water, THF, acetonitrile and ethyl acetate, as shown in Figure 7. It is observed that, in the case of aprotic-polar solvents, the conversion of the substrates and the selectivity of the product decreases compared to the protic solvents. The accumulation of N-(substituted-phenyl)-hydroxylamine intermediaries were detected reaching selectivities of ~5% for both NFMF and NFMO substrates when THF was used as solvent. However, in the case of protic solvents, higher reaction rates and a very low accumulation of reaction intermediates (<1%) are detected. 

These results suggest that the protic solvents favour both activity and selectivity in the hydrogenation reaction, whereas aprotic-polar solvents showed an opposite trend. This behaviour could be explained considering the proposed Haber reaction mechanism, in which the rate-limiting step involves the hydrogenation of N-phenyl-hydroxylamine derivate to the corresponding aniline, as shown in Scheme 3. A growing electron density on the N atom is expected to occur in the transition state of this rate-determining step [61]. We suggest the presence of protic solvents can stabilize the transition state, thus accelerating the hydrogenation of N-(substituted-phenyl)-hydroxylamine intermediaries, to produce the corresponding anilines in comparison with aprotic-polar solvents. 

Although complete conversion and selectivity (>99%) was obtained in the presence of water as a reaction medium for the hydrogenation of both NFMF and NFMO, the conversion followed the order ethanol < methanol < water. However, despite the solvent, the corresponding anilines selectivity ranged between 98% and 99.5%. The use of water as a solvent in reaction was found to be most effective in the hydrogenation of these nitroarenes compared to the other solvents. 

#### 3.2.3. Reusability and Operational Stability of the Catalyst

**Figure 8:**
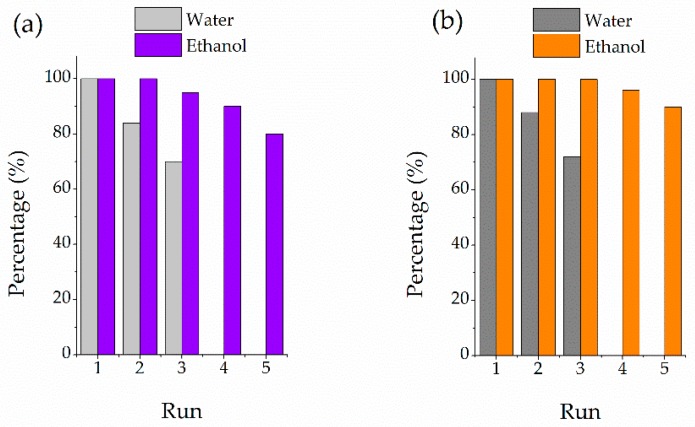
Recycling studies in the hydrogenation of (**a**) NFMF and (**b**) NFMO at 180 min of reaction using water and ethanol as solvent.

In order to assess both reuse capacity and the operational stability of the 0.5Au/ANW catalyst, water and ethanol were chosen as solvents, as shown in Figure 8. It is observed that the catalyst shows different operational stability depending on the solvent used. In the case of water, a continuous loss of conversion is detected, reaching a maximum conversion around 70% at the third operating cycle in both substrates evaluated. For the use of ethanol, it can be observed that it reaches higher operational stability, reaching 80% conversion in the fifth operating cycle. After each operation cycle, AAS characterization of the supernatant was performed to evaluate the metal leachate between operation cycles. 

In the case of the use of water recycling, a leaching of about 46% of the metal was detected at the end of the three operating cycles as shown in Table S1 in the Supplementary Material. Under the operating conditions, in an aqueous medium, hydrothermal reactions on the catalyst surface that promote the leaching of the active phase to the reaction medium are favoured [62,63,64]. However, when ethanol is used as a solvent, only about 8% metal leaching was detected after the fifth operating cycle. These results correlate directly with the stability shown by the support in aqueous medium. 

## 4. Conclusions

It was possible to synthesize heterogeneous catalysts based on Au nanoparticles supported in Al_2_O_3_ nanowires that were active, selective and with moderate operational stability, in the hydrogenation of nitroarenes of pharmaceutical interest. The catalytic properties were controlled with the metal loading on the supports where it was found that, with a nominal content of 0.50% mass of Au, an optimal system for the reaction of interest was obtained. The effect of the solvent plays an important role in the activity and selectivity of the catalyst, where the highest efficiency and operational stability was achieved when ethanol is used as the solvent. Five consecutive cycles were achieved using ethanol, reaching 80% conversion in the last run for the best catalytic systems. 

## Figures and Tables

**Table 1:** Physicochemical characterization data obtained by atomic absorption spectrometry (AAS), N_2_ isotherms and transmission electron microscopy (TEM) for the pristine ANW support, ANW-NH_2_ and xAu/ANW catalysts.

Material	Au Loading (%)	S_BET_ (m^2^g^−1^)	ANW d_pore_ (nm)	Au d_TEM_ (nm)
ANW	-	204	8.9	-
ANW-NH_2_	-	154	8.5	-
0.25Au/ANW	0.21	138	8.0	3.7 ± 1.8
0.50Au/ANW	0.42	126	8.1	4.0 ± 1.1
1.00Au/ANW	0.87	115	7.9	4.6 ± 2.6

**Table 2:** Binding energies (BE) of internal levels and atomic ratio of (x)Au/ANW catalysts. (Percentage in brackets).

x	Al 2p (eV)	Au 4f_7/2_ (eV)		O 1s (eV)	Au/Al at
		Au^0^	Au^δ+^		
0.25	74.5	83.6 (91)	84.8 (9)	531.4	0.022
0.50	74.5	83.8 (84)	85.0 (16)	531.3	0.031
1.00	74.4	84.0 (78)	85.1 (22)	531.3	0.009

## Supplementary Materials

The following are available online at https://www.mdpi.com/1996-1944/13/4/925/s1.
