# Supplementary material for: Liquid Phase Hydrogenation of Pharmaceutical Interest Nitroarenes over Gold-Supported Alumina Nanowires Catalysts
Source: Materials (Basel). 2020 Feb 19;13(4):925. doi: 10.3390/ma13040925 (PMC7078662; doi:10.3390/ma13040925)
Supplement: Supplementary file 1 [file materials-13-00925-s001.pdf]

materials

MDPI

Supplementary Material

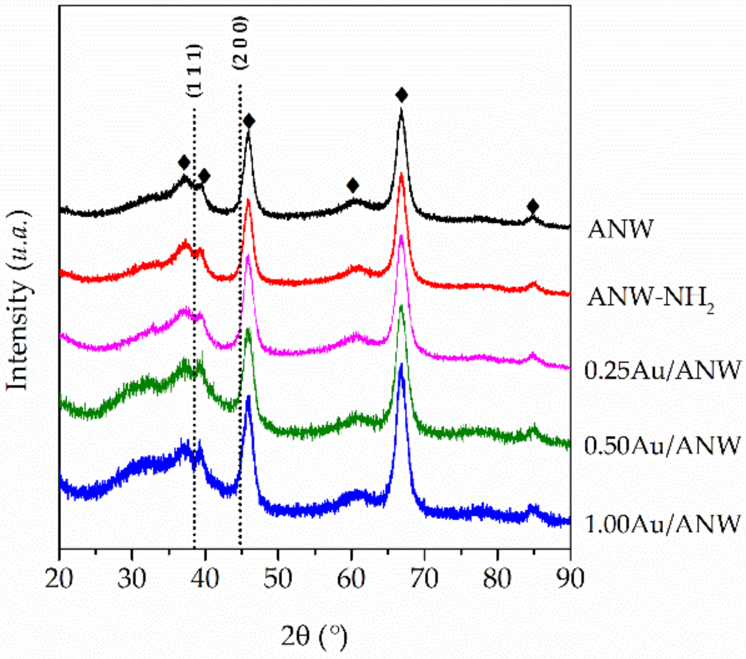

Figure S1. X-ray diffractograms for prepared materials. ♦ γ-Al_2O_3 (JCPDS 86-1410) and dotted line corresponds to the diffractions for metallic Au (JCPDS: 21-1272).

Table S1. AAS characterization during the recycles studies.

Solvent	Run	Au supernatant (ppm)	Leaching (%)[(a)]
Water	1	294	7.1
	2	588	14.5
	3	1050	24.6
	Total	1932	46.1
Ethanol	1	8.4	0.2
	2	29.4	0.7
	3	46.2	1.1
	4	88.2	2.1
	5	164	3.9
	Total	336	8.0

[(a)] The total of gold loading used from AAS characterization for the catalysts.
